# Inducing factors and deformation mechanism of the Zhangjiacitang landslide in the Three Gorges Reservoir Area

**DOI:** 10.1038/s41598-023-40186-6

**Published:** 2023-08-09

**Authors:** Hongyu Chen, Jianhua Zou, Xinghua Wang, Peng Lv, Zefu Tan, Longfei Cheng, Qiang Wei

**Affiliations:** 1 Sustainable Development Center of the Three Gorges Reservoir Area, Chongqing Three Gorges University, Chongqing, 404020 China; 2 School of Civil Engineering, Chongqing Three Gorges University, Chongqing, 404020 China; 3 Sichuan Geological Environment Survey and Research Center, Chengdu, 610081 China; 4 Institute of Geographic Sciences and Natural Resources Research, Chinese Academy of Sciences, Beijing, 100101 China

**Keywords:** Natural hazards, Civil engineering, Environmental impact

## Abstract

Landslides are the most widely distributed geological hazards in the Three Gorges Reservoir Area (TGRA). Understanding the deformation mechanism and evolution of landslides is of great significance for their prevention and control. In this study, we focused on the Zhangjiacitang landslide, a typical bank landslide in the TGRA. We analyzed the relationship between landslide deformation and water level fluctuations and rainfall, based on accumulated displacement monitoring data, to clarify their triggering factors and deformation mechanism. The results show that the Zhangjiacitang landslide is a large-scale accumulation landslide. Under the influence of cyclic water level fluctuations and periodic rainfall, the accumulated displacement–time curve shows a “stepped” characteristic. Heavy rainfall emerged as the primary factor influencing the deformation of the Zhangjiacitang landslide, leading to substantial deformation throughout different periods. The deformation of the landslide exhibited a positive correlation with the intensity of rainfall. In contrast, the impact of water level changes on the landslide deformation was more intricate. A rapid water level drop (> 0.3 m/d) tended to intensify the landslide deformation, while the slow water level drop period (< 0.3 m/d) did not exhibit such an effect. This study emphasizes the need for closely monitoring the landslide status during heavy rainfall periods and rapid water level decline periods. The findings of this study provide a certain reference for landslide monitoring, early warning, prevention and control in the TGRA.

## Introduction

Landslide deformation is a complex geological process that is controlled not only by the geological environment such as geological structure, lithology, and topography, but also by various inducing factors such as rainfall, reservoir water level fluctuations, and human engineering activities^1–5^. It has been commonly observed that bending deformation occurs on the anticline rock slopes in the Wuxia section of the TGRA^6^. This phenomenon is primarily attributed to the presence of an upper hard and lower soft sedimentary structure, as well as the rapid down-cutting of the river valley^6^. The main controlling factors of the Shanshucao landslide are the interphase lithologic assemblage and the developed joint fissures, while heavy rainfall is a necessary condition for the landslide deformation to occur^7^. The deformation of the Muyubao landslide is influenced by topography, geomorphology, stratigraphic lithology, and geological structure, with the reservoir water level serving as the main driving factor^8^. The Tanjiahe landslide produced deformation during 2008–2014 mainly concentrated in the high-water level operation period, which was caused by the soaking and softening effect of the rising reservoir water level^9^.

Since the construction of the Three Gorges Project, reservoir impoundment has disrupted the geological balance of the reservoir banks, making them susceptible to deformation under the influence of fluctuating reservoir water levels and rainfall^10,11^. Water-related landslides can be classified into four main types based on their inducing factors: buoyancy weight loss landslides, hydrodynamic pressure landslides, rainfall landslides, and mixed-type landslides^12,13^. Buoyancy weight loss landslides experience significant deformation during the rising period of the reservoir water level, while deformation tends to decrease during the falling period, such as the Muyubao landslide^8,14^. Hydrodynamic pressure landslides experience significant deformation during the falling period of the reservoir water level, and the greater the falling speed, the more significant the deformation, such as the Bazimen landslide and Baijiabao landslide^15–18^. The extent of deformation in rainfall landslides is significantly correlated with rainfall intensity and type. For example, this relationship is observed in the Tanjiahe landslide and the Shiliushubao landslide^19,20^. For mixed-type landslides, their deformation is significantly related to the changes in reservoir water level and rainfall, such as the Sanzhouxi landslide and the Outang landslide^21,22^.

Studying the influence and interrelationships between reservoir water level fluctuations, rainfall, and reservoir bank landslide deformation characteristics has important theoretical and engineering significance. Comprehensive monitoring of bank slope landslides is essential for acquiring on-site data and establishing a fundamental basis for a more thorough analysis^23–27^. Since the beginning of the 21st century, the development of geological hazard monitoring technology has become more accurate, faster, and more automated^28^. Commonly used landslide monitoring techniques mainly include surface displacement monitoring, deep displacement monitoring, groundwater level monitoring, and rainfall monitoring, among which displacement monitoring changes directly reflect the macroscopic phenomena of landslide deformation and indirectly reflect the changes of various indicators inside the slope in different stages of deformation^29,30^. Therefore, many scholars employ landslide monitoring technology to integrate real-time monitoring data with field site investigations and numerical simulations for the study of factors and causal mechanisms inducing landslide deformation, primarily through qualitative analysis^25,31–33^. However, only a few studies have determined the main controlling factors of landslide deformation through quantitative analysis and subsequently analyzed the landslide's triggering mechanism by combining the displacements of monitoring points with the dynamic processes of reservoir water level changes and rainfall patterns.

Based on long-term real-time monitoring data from the Zhangjiacitang landslide in the TGRA, this study conducted a detailed analysis by categorizing the variations in reservoir water level based on the water level dispatch of the Three Gorges Reservoir. The study statistically analyzed the deformation rates of the landslide, changes in reservoir water level, rainfall, and other related parameters during different periods of landslide deformation. By employing quantitative process analysis methods, the study investigated the degree of influence on landslide deformation under the conditions of rainfall and reservoir water level changes, and elucidated the inducing factors and deformation mechanism.

## Review of the Zhangjiacitang landslide

**Figure 1:**
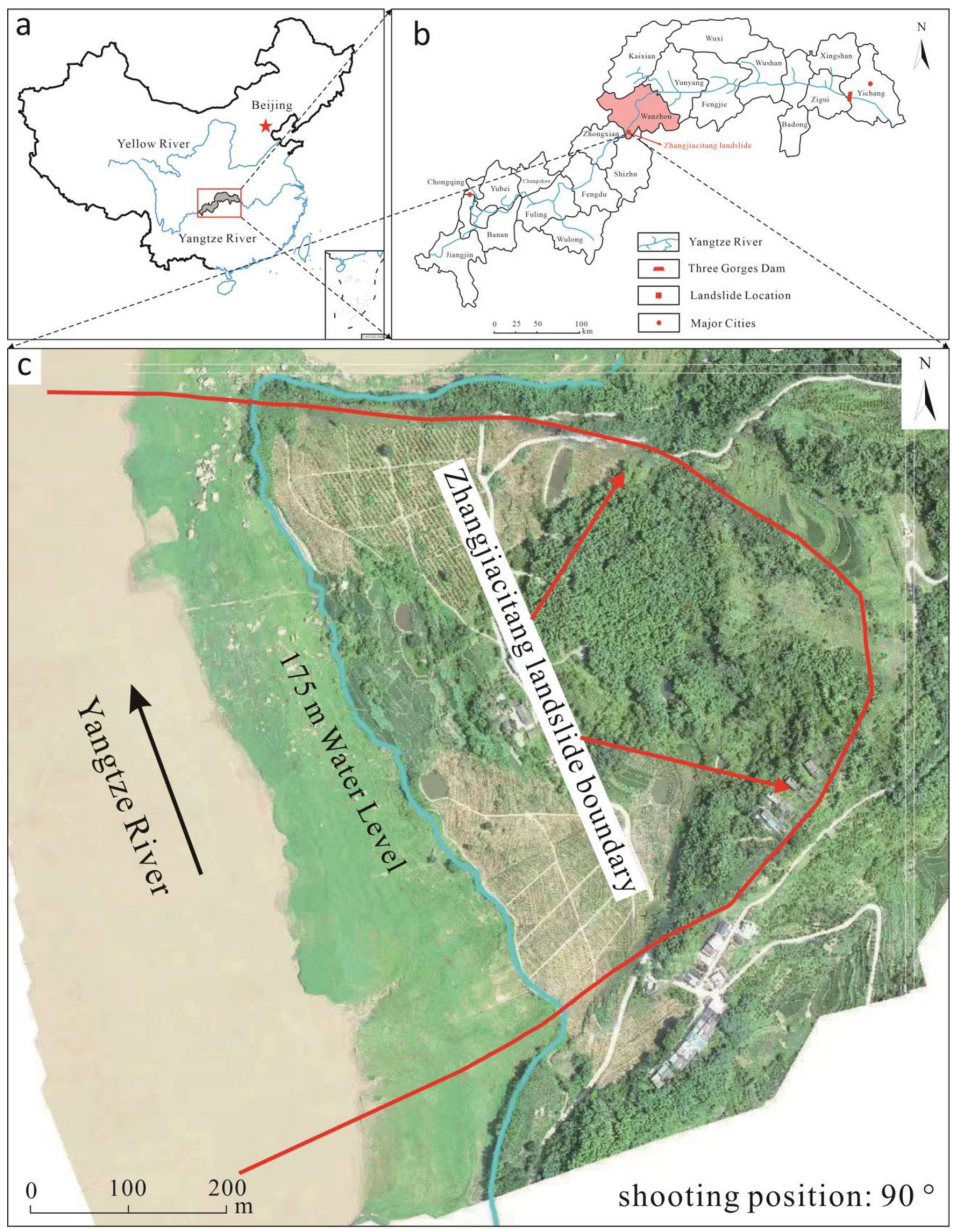
Location of the Zhangjiacitang landslide (**a**, **b**)^34^ and its geomorphological delimitation (**c**) (remoting sensing image come from https://www.91weitu.com). (This figure is edited and generated by CorelDraw2020 software).

The Zhangjiacitang landslide is located on the right bank of the Yangtze River in Xinxiang Town, Wanzhou District, TGRA in Chongqing, China (Fig. 1), with a longitude of 108° 15′ 7″ and a latitude of 30° 28′ 41″. The landslide area exhibits a tectonic denudation slope landform, with a gradual decrease in terrain from east to west. The overall topography in the area is characterized by gentle slopes, with slightly steeper sections at the rear and front, and a slightly milder slope in the central part. The average slope angle ranges from 10 to 20°, and the slope direction is approximately 249°. The back edge of the slope where the landslide is located is characterized by a sandstone cliff, which is nearly vertical and has a height of approximately 30 m. The middle and back portions of the slope have a gradient of 10–15° and are primarily covered by bamboo forest. The central part of the landslide exhibits a gentler slope with an inclination of 5–10°, featuring residential houses and cultivated land. The front section of the landslide displays a distinct terrace-like landform, with a slope ranging from 15 to 20°, predominantly consisting of agricultural and cultivated land.

The region is characterized by unique natural conditions, being situated in a subtropical monsoon warm and humid climate zone with abundant rainfall. The average annual temperature is 18.1°C, and there is a noticeable temperature variation between the river valley and the surrounding mountains, with the river valley being 1–3 °C warmer. The average annual rainfall is 1191.3 mm, with the highest recorded annual rainfall reaching 1635.2 mm. Rainfall is predominantly concentrated between May and September, accounting for approximately 60–70% of the total annual rainfall. Spring and summer are particularly prone to heavy rainstorms, with daily rainfall often exceeding 100 mm and the longest continuous rainfall lasting for 16 days. Based on 37 years of meteorological data, the historical record for the highest daily rainfall stands at 243.31 mm, which occurred on July 16, 1982.

The landslide area is situated in the southeastern part of the northeastern section of the Fengdu-Zhongxian oblique. The rock layers exhibit monoclinic outcrop, with a rock layer inclination of 240° and a dip angle of 13°. There are no faults traversing the area, and the slope shows a downward trend. In terms of Quaternary deposits, the area primarily consists of Holocene Colluvial Deposits (Q_4_^col + dl^), landslide Accumulation (Q_4_^del^), Quaternary Residual Slope Deposit (Q_4_^el + dl^), and Quaternary Alluvial (Q_4_^al + pl^). The predominant bedrock stratum exposed in the area is the Middle Jurassic Lower Shaximiao Formation (J_2_xs). The Zhangjiacitang landslide material comprises predominantly of blocky gravelly soil, powdery clay, blocky soil, and isolated boulders. The sliding belt soil mainly consists of gravelly clayey silt with rock fragments. The sliding bed belongs to the middle Jurassic Shaximiao Formation (J_2_s), primarily consisting of strongly to moderately weathered mudstone with locally distributed sandstone, and the rock mass is relatively intact with a dip direction of 238° and a dip angle of approximately 13°.

**Figure 2:**
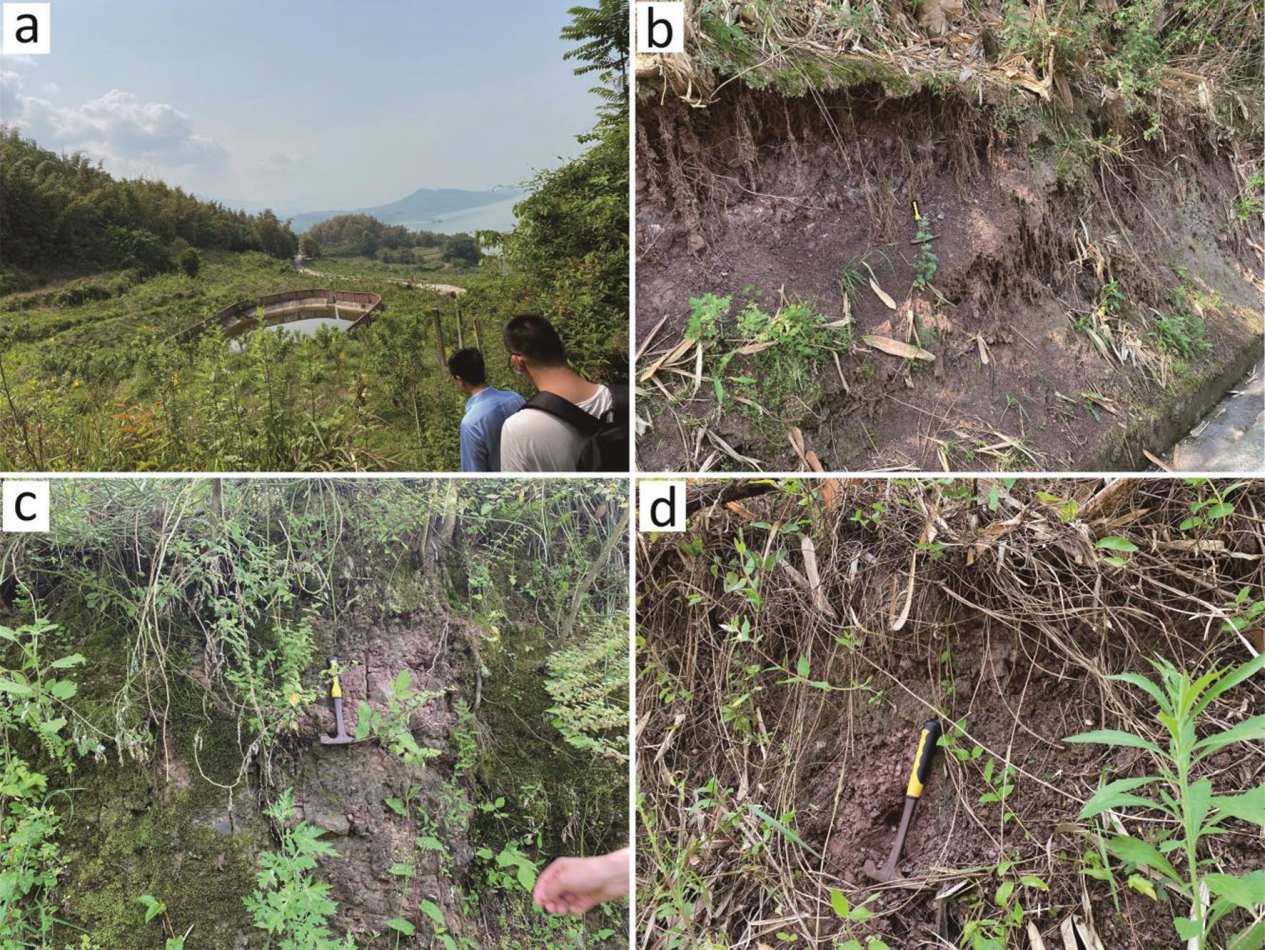
Field images showing the boundaries of the Zhangjiacitang landslide. (**a**) left boundary; (**b**) right boundary; (**c**) left trailing edge boundary; (**d**) right trailing edge boundary.

**Figure 3:**
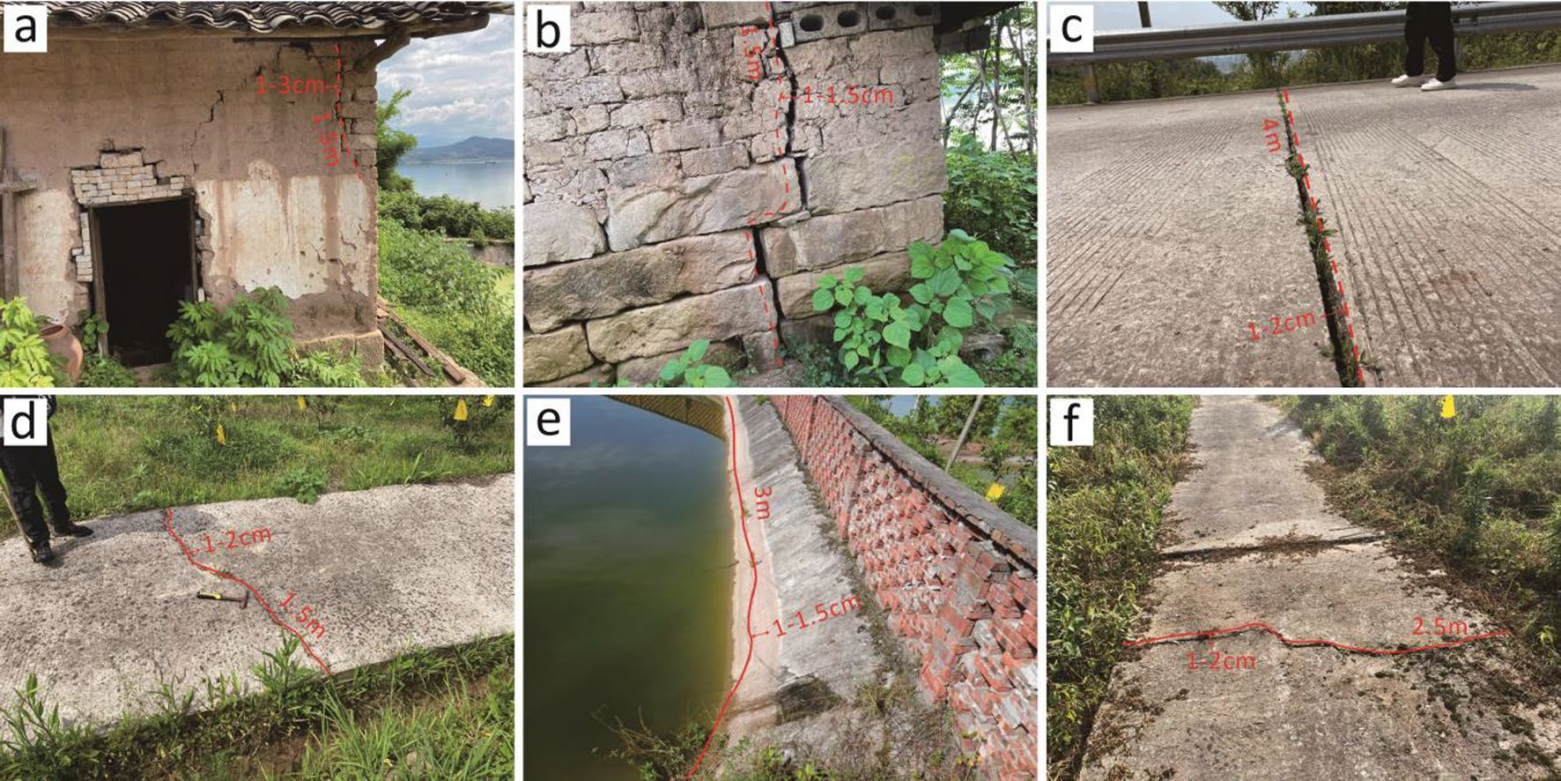
Photographs of the deformation site of the Zhangjiacitang landslide. (**a**–**c**) deformation of the left side in the central part of the landslide; (**d**) deformation of the right side in the central part of the landslide; (**e**) deformation of the left side in the rear part of the landslide; (**f**) deformation of the left side in the front part of the landslide.

The overall morphology of the Zhangjiacitang landslide is tongue-shaped, with the ridge on the left side and bedrock outcrops on the right side. The front edge of the landslide advances into the Yangtze River with an elevation of about 130 m, while the rear edge is bounded by bedrock outcrops with an elevation of 240–250 m (Fig. 2). The vertical difference between the front and rear edges is approximately 110–120 m. The Zhangjiacitang landslide measures about 650 m in width and 500 m in length, with an average thickness of 20 m, an area of approximately 32.5 × 10^4^ m^2^, and a volume of around 650 × 10^4^ m^3^. The main sliding direction of the landslide is 249°, and it belongs to a large-scale accumulation landslide related to water. According to the site investigation and the information collected, the Zhangjiacitang landslide has undergone significant deformation in recent years, mainly concentrated in the central left side of the landslide (Fig. 3). If the landslide were to flow into the Yangtze River, it would threaten the normal operation of shipping on the river. For this reason, researchers and local authorities have carried out deformation monitoring in recent years.

## Method

The monitoring of the Zhangjiacitang landslide involved surface deformation monitoring and rainfall monitoring. For surface displacement, GPS instruments were selected as automatic monitoring instruments, while automatic rain gauges were used for rainfall monitoring. These instruments were configured to collect, transmit, and process data at specified monitoring intervals (Table 1, Supplementary material). All collected data was consistently uploaded to the data center for analysis.

**Table 1:** Main technical indicators of monitoring instruments.

Monitoring Methods	Monitoring Instruments	Monitoring mode	Precision	Frequency of monitoring
Surface displacement monitoring	GPS	Real-time	Horizontal: ± 2.5 mm + 1ppmRMS Vertical: ± 5 mm + 1 ppm RMS	Every 1 h
rainfall monitoring	Tumbler type Rain gauge	Real-time	0.1 mm	Every 5–60 min

**Figure 4:**
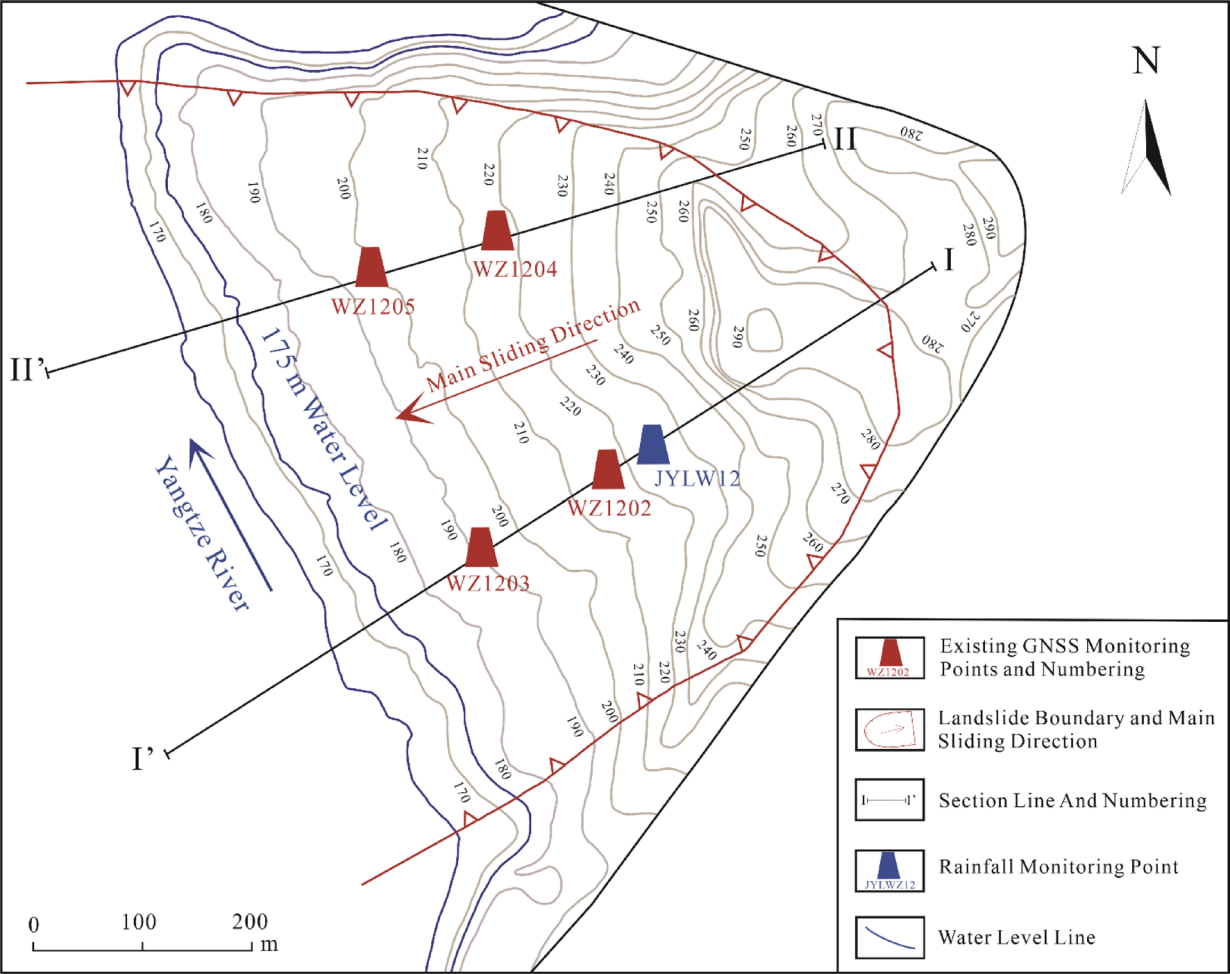
Distribution of monitoring points in the Zhangjiacitang landslide. (This figure is edited and generated by CorelDraw2020 software).

**Figure 5:**
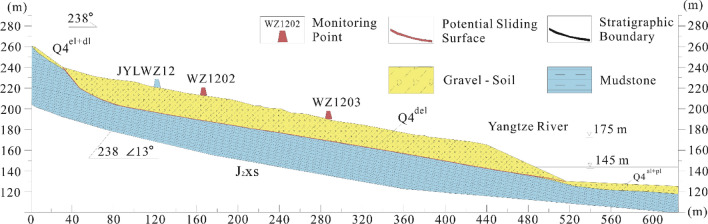
I–I′ engineering profile of the Zhangjiacitang landslide.

The Zhangjiacitang landslide is monitored by two profiles on the east and west sides, labeled I–I′ and II–II′, respectively, as shown in Fig. 4. One deformation monitoring point is installed at the upper and lower parts of each profile, and one rainfall monitoring point (JYLWZ12) is also installed on the I–I′ profile as shown in Fig. 5. The reservoir water level changes using the reservoir water level dispatch data provided by Wanzhou District Geological and Environmental Monitoring Station. Since the deformation of landslide II–II′ profile is weak, this study focuses on the GNSS cumulative horizontal displacement data, rainfall, and reservoir water level data collected from June 1, 2019 to December 31, 2021 at the west side profile (I–I′). These data are analyzed to understand the deformation behavior of the landslide.

According to the water level scheduling in the TGRA, the variations in water level can be categorized into five periods: a slow decline period from January to April (average decline rate < 0.3 m/d), a rapid decline period from May to June (average decline rate > 0.3 m/d), a low water operation period in July and August, a water level rising period in September and October, and a high-water operation period in November and December. During the flood season from June to September, the reservoir water level undergoes frequent adjustments and experiences significant fluctuations. The study area experiences concentrated rainfall from May to September, with distinct autumn rain occurring in October. It is evident that the occurrence of abrupt deformation in landslides is closely related to the periodic variations of reservoir water level and rainfall.

This study classified the landslide deformation into two stages based on the displacement deformation rate: the strong deformation stage (average displacement increment > 0.15 mm/d, reaching a maximum of 0.57 mm/d) and the no significant deformation stage (average displacement increment < 0.05 mm/d). Single-factor analysis was conducted to investigate the individual effects of reservoir water level changes and rainfall on landslide deformation, as well as the combined effects of these two factors. Considering the varying impacts of reservoir water level changes in different stages, the process of reservoir water level changes was carefully divided during the strong deformation stage and the no significant deformation stage, excluding the influence of rainfall. The relationship between landslide deformation and the decrease, increase, high water operation, and low water operation of reservoir water level was determined, and the degree of their influences was quantified. Furthermore, taking into account the effects of rainfall intensity, cumulative rainfall, and other factors, the relationship between landslide deformation and rainfall was analyzed using the monitored rainfall data in the landslide area, while disregarding the influence of reservoir water level changes. Finally, by statistically analyzing parameters such as landslide deformation rate, reservoir water level change rate, and rainfall, the impacts of rainfall on landslide deformation under different reservoir water level change stages were compared. The study conducted a dynamic analysis of the coupling effects between reservoir water level and rainfall, revealing the primary and secondary influencing factors of landslide deformation under the combined action of reservoir water level and rainfall.

## Results

### Characteristics of the monitoring data

**Figure 6:**
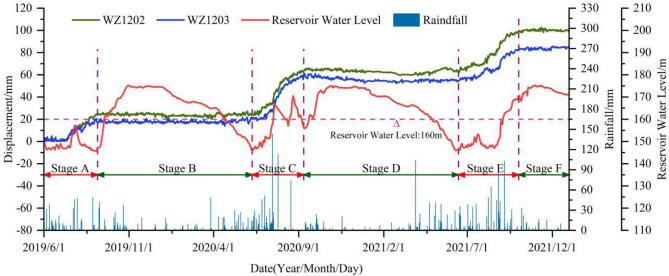
Cumulative surface horizontal displacement monitoring curves of I–I′ section in the Zhangjiacitang landslide.

The cumulative displacement curves of monitoring points WZ1202 and WZ1203 exhibit a "step-like" pattern, which is consistent with the characteristics of bank landslides in the TGRA^34–37^. Under the coupled influence of water level and rainfall, the Zhangjiacitang landslide experienced continuous and significant displacement during certain periods (average displacement growth rate > 0.15 mm/d, up to 0.57 mm/d), indicating a strong deformation phase (Fig. 6, stages A, C, and E). In contrast, during other periods, the cumulative displacement tended to stabilize (average displacement growth rate < 0.05 mm/d) without apparent deformation (Fig. 6, stages B, D, and F), exhibiting a periodic characteristic in a stair-like pattern.

**Figure 7:**
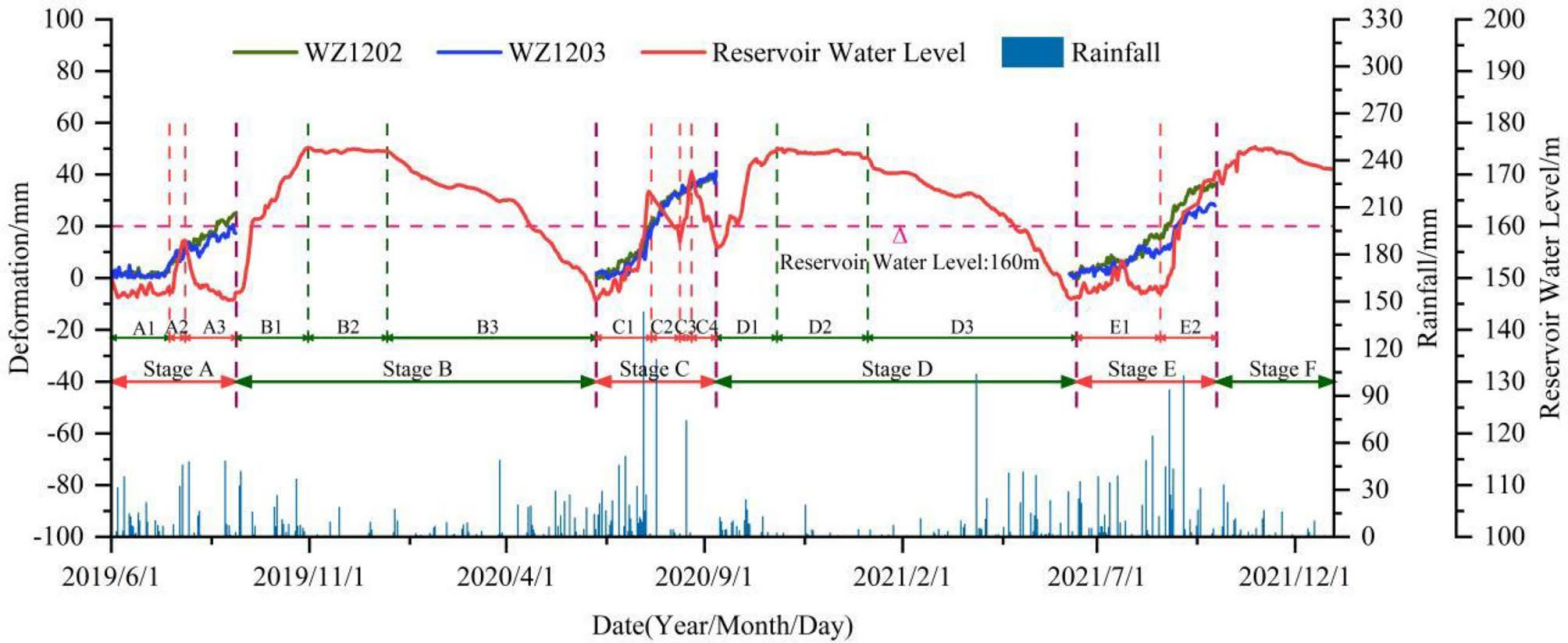
Cumulative displacement of parts A, C, and E during the strong deformation stages.

From June 1, 2019 to December 31, 2021, the cumulative surface horizontal displacement of the rear monitoring point WZ1202 and the front monitoring point WZ1203 of the Zhangjiacitang landslide were 100.74 mm and 85.36 mm, respectively, with a difference in cumulative deformation of 15.38 mm, and the difference gradually increased over time (Fig. 6). The deformation directions of the two points were 209° and 221°, respectively. The average monthly rainfall from June to September 2019 (Stage A), mid-June to early September 2020 (Stage C), and mid-June to October 2021 (Stage E) were 148 mm, 247 mm, and 233 mm, respectively. The average cumulative displacement increments of the two monitoring points were 23 mm, 40 mm, and 30 mm, respectively, with average displacement rates of 0.24 mm/d, 0.43 mm/d, and 0.28 mm/d (Fig. 7). The average monthly rainfall from September 2019 to early June 2020 (Stage B), mid-September 2020 to early June 2021 (Stage D), and from October to the end of December 2021 (Stage F) were 75 mm, 78 mm, and 59 mm, respectively. It can be concluded that the average monthly rainfall during the strong deformation stage was much greater than during the stage with no obvious deformation, with Stage C having the highest rainfall, cumulative displacement increment, and displacement rate.

### Responses of landslide deformation to reservoir water level and rainfall

The relationship between the average displacement monitoring data of monitoring points WZ1202 and WZ1203 and the reservoir water level and rainfall are shown in Tables 2 and 3.

**Table 2:** Statistics of average displacement increment, displacement growth rate and related parameters of monitoring points WZ1202 and WZ1203 during the strong deformation stages.

Deformation period	Time period (Year/Month/Day)	Average displacement increment (mm)	Average displacement growth rate (mm/d)	Reservoir water level change (m)	Reservoir water level change rate (m/d)	Cumulative rainfall (mm)	Maximum daily rainfall (mm/d)	Average daily rainfall (mm/d)
Stage A								
A1	2019/6/1–2019/7/19	7.5	0.15	146 ~ 148	/	218.0	38.5	4.45
A 2	2019/7/20 – 2019/7/28	4.5	0.50	147 → 157	+ 1.11	79.9	46	8.88
A3	2019/7/29 – 2019/8/31	10.0	0.29	157 → 146	− 0.32	145.5	48.5	4.28
Stage C								
C1	2020/6/11 – 2020/7/22	19.5	0.46	146 → 167	+ 0.50	534.1	143.4	12.72
C2	2020/7/23 – 2020/8/13	12.5	0.57	167 → 157	− 0.45	127.8	113.4	5.81
C3	2020/8/14 – 2020/8/22	4.0	0.44	157 → 170	+ 1.44	78.2	74.3	8.69
C4	2020/8/23 – 2020/9/10	4.0	0.21	170 → 156	− 0.74	0.5	0.5	0.03
Stage E								
E1	2021/6/11 – 2021/8/19	11.5	0.16	146 ~ 153	/	428.0	64.6	6.11
E2	2021/8/20 – 2021/9/30	18.5	0.44	147 → 170	+ 0.55	412.3	102.9	9.82

+ , the reservoir water level rises; −, the reservoir water level drops; /, no significant change.

**Table 3:** Statistics of related monitoring parameters of monitoring points WZ1202 and WZ1203 during no significant deformation stages.

Deformation period	Time period (Year/Month/Day)	Reservoir water level change (m)	Reservoir water level change rate (m/d)	Cumulative rainfall (mm)	Maximum daily rainfall (mm/d)	Average daily rainfall (mm/d)
Stage B						
B1	2019/9/1 – 2019/10/31	146 → 175	+ 0.48	250.9	42.0	4.11
B2	2019/11/1 – 2019/12/31	175	/	47.5	19.0	0.78
B3	2020/1/1 – 2020/6/10	175 → 146	− 0.18	400.7	49.1	2.47
Stage D						
D1	2020/9/11 – 2020/10/27	156 → 175	+ 0.40	156.2	23.8	3.32
D2	2020/10/28 – 2021/1/5	175	/	47.2	20.5	0.67
D3	2021/1/6 – 2021/6/10	175 → 146	− 0.19	501.8	103.9	3.22
Stage F						
	2021/10/1 – 2021/12/31	175	/	175.8	16.9	1.93

+ , the reservoir water level rises; −, the reservoir water level drops; /, no significant change.

During the strong deformation stage (Fig. 7, Table 2), stages A2, C1, C3, and E2 were in the rise period of reservoir water level accompanied by heavy rainfall (with an average daily rainfall of 8.69–12.72 mm/d), with average displacement increments of 4.5 mm, 19.5 mm, 4.0 mm, and 18.5 mm, and average displacement rates of 0.50 mm/d, 0.46 mm/d, 0.44 mm/d, and 0.44 mm/d, respectively. Stages A3, C2, and C4 were in the rapid decline period of reservoir water level (with an average decline rate > 0.3 m/d). Among them, stages A3 and C2 were accompanied by strong rainfall (with an average daily rainfall of 4.28–5.81 mm/d), with average displacement increments of 10.0 mm and 12.5 mm, and average displacement rates of 0.29 mm/d and 0.57 mm/d, respectively. Stage C4 had small rainfall (with an average daily rainfall of about 0.03 mm/d), with an average displacement increment of 4.0 mm and an average displacement rate of 0.21 mm/d. Stages A1 and E1 were in the low water level high-frequency low amplitude period, accompanied by strong rainfall (with an average daily rainfall of 4.45–6.11 mm/d), with average displacement increments of 7.5 mm and 11.5 mm, and average displacement rates of 0.15 mm/d and 0.16 mm/d, respectively.

During the stage of no obvious deformation (Fig. 7, Table 3), stages B1 and D1 were in the rise period of reservoir water level, stages B3 and D3 were in the slow decline period of reservoir water level (with an average decline rate < 0.3 m/d), and there was no obvious displacement change at the monitoring points. Stages B2, D2, and F were in the high-water level period (about 175 m), and this period was not during the flood season, with small rainfall (0.67–1.93 mm/d).

## Discussion

### Analysis of landslide inducing factors

By analyzing the monitoring data of the Zhangjiacitang landslide (Fig. 6), it can be concluded that the displacement change at the monitoring point WZ1203 in the front of the landslide is smaller than that at WZ1202 in the rear, and the displacement change and deformation direction of the front and rear monitoring points are essentially synchronous, indicating that the landslide is translational and has overall characteristics. The period from June to September (flood season) each year is the "active period" of the landslide, during which the landslide undergoes strong deformation with rapid displacement growth. Based on the displacement monitoring data, along with the reservoir water level and rainfall data, it can be inferred that the "active period" of the landslide is characterized by abundant rainfall and the reservoir water level is at a middle-low level, whereas the "dormant period" of the landslide is characterized by less rainfall and the reservoir water level is often at a middle-high level. This implies that the deformation of the Zhangjiacitang landslide is closely related to periodic rainfall and changes in reservoir water level.

### Reservoir water level fluctuations

**Figure 8:**
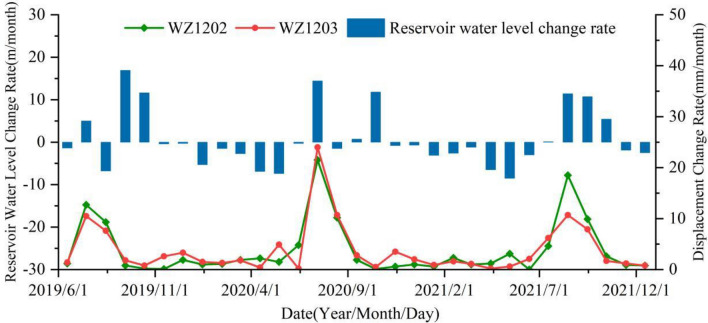
The relationship between reservoir water level change rate and displacement rate.

**Figure 9:**
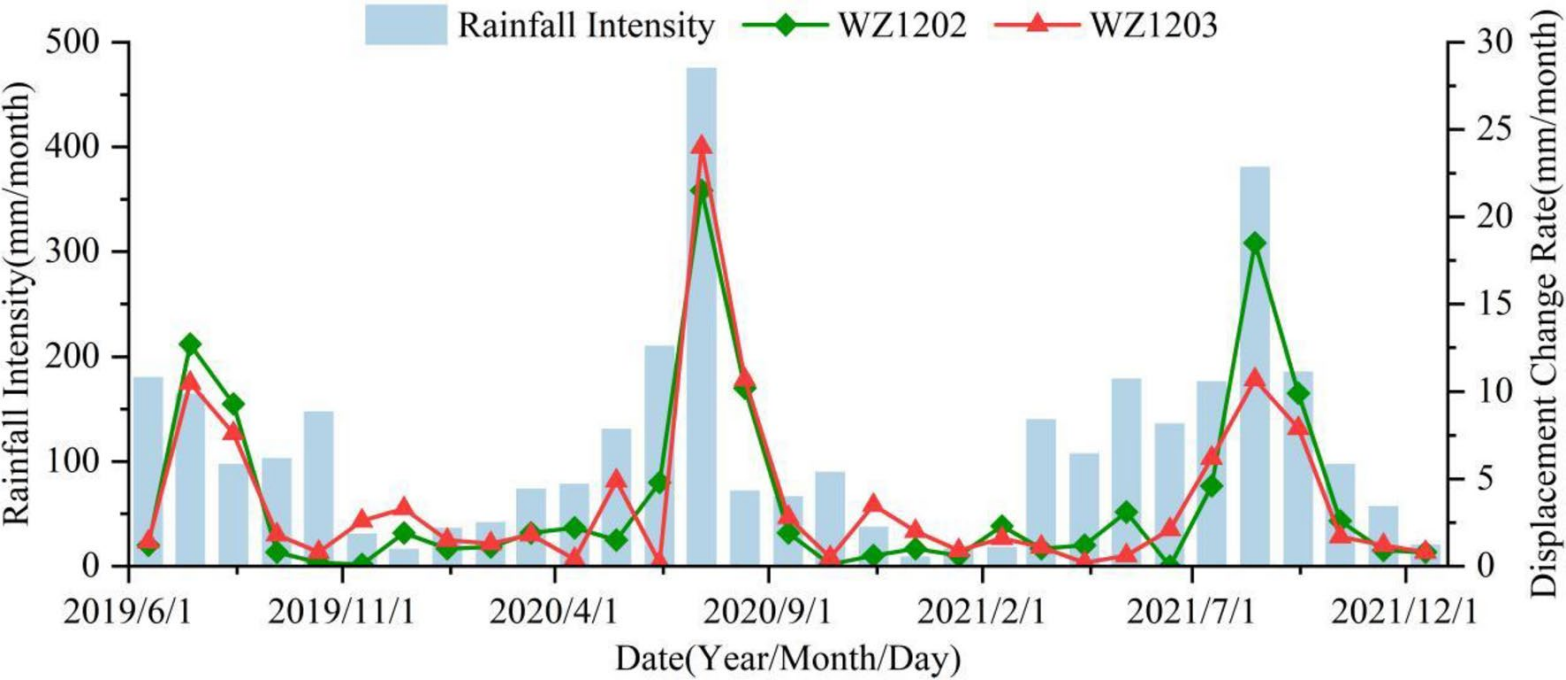
The relationship between average rainfall intensity and average rate of displacement change.

In this study, the relationships between reservoir water level fluctuations, rainfall intensity, and displacement deformation rates of the Zhangjiacitang landslide from 2019 to 2021 are revealed in Figs. 8 and 9. The reservoir water level fluctuates within the medium-low water level from June to August, reaches the highest water level in September and October, and slowly declines to the lowest water level from January to June of the following year (Fig. 8). The maximum and minimum displacement deformation rates differ significantly, with WZ1202 ranging from 0.1 to 21.5 mm/month and WZ1203 ranging from 0.2 to 24.0 mm/month. The acceleration stage of deformation occurs in June (Table 2), and the peak values of displacement deformation rates are distributed in July and August each year, which coincides with the frequent fluctuation period of the reservoir water level in the medium-low water level and the possible triggering of seasonal heavy rainfall, indicating that the reservoir water level fluctuations may not be the sole cause of the observed changes. In addition, from January to May each year, which is the non-flood season with small rainfall intensity, the displacement deformation is accompanied by the decline of the reservoir water level. The greater the rate of the reservoir water level decline, the greater the displacement deformation rate. In contrast, from September to October each year, the reservoir water level gradually rises at a relatively high rate, but the displacement deformation rate is relatively small compared to the non-flood season. Therefore, it can be concluded that the reservoir water level decline has a greater impact on the deformation of the landslide compared to the rise of the reservoir water level.

#### Rainfall

The study area experiences the majority of its annual rainfall between May and September, with sustained or short-term heavy rainfall events. Without considering the change of the reservoir water level, the trend of displacement change rate is found to be in accordance with that of the rainfall intensity. That is, an increase in rainfall intensity leads to a corresponding increase in the displacement change rate, and vice versa, indicating a positive correlation (Fig. 9). The peak values of displacement change rate occur during the periods of heavy rainfall, such as in July 2020, when the displacement change rates of WZ1202 and WZ1203 reached the maximum, and the rainfall intensity also peaked at 475.6 mm/month during this period. Hence, it can be concluded that heavy rainfall is a significant factor that triggers landslide acceleration deformation.

#### Coupling effects of rainfall and reservoir water level fluctuation

Under the condition of not considering the influence of rainfall, the decrease of water level can induce landslide deformation, and heavy rainfall has a significant effect on landslide deformation without considering the change in water level. However, bank slope landslides generally undergo deformation under the coupling effect of water level and rainfall, and the relationship between water level, rainfall, and landslide deformation needs to be further investigated. The following section provides a further analysis of the impact of rainfall accompanying different stages of water level on landslides.

- The influence of rainfall on landslide during reservoir water level rising

**Figure 10:**
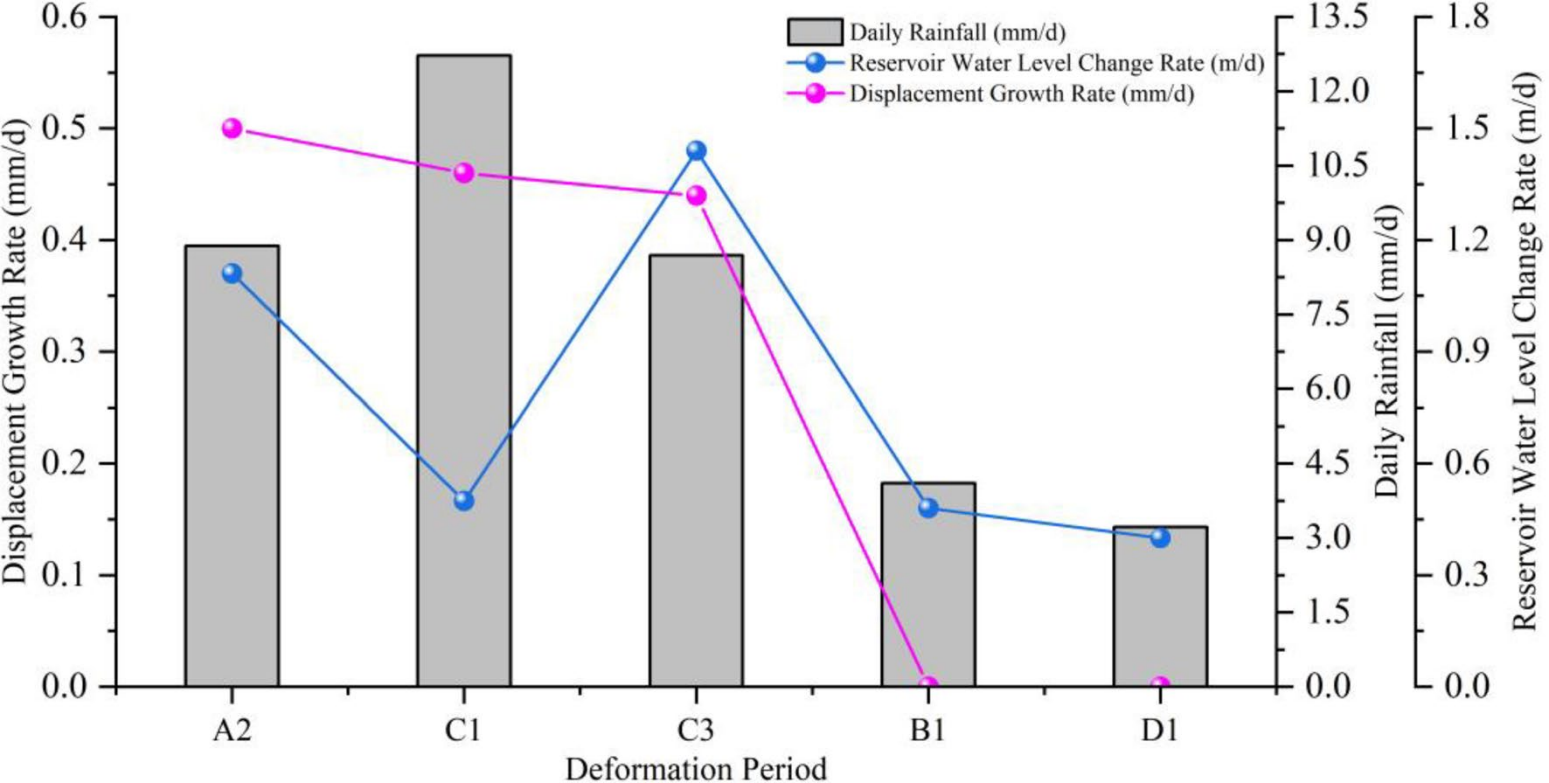
The relationship between landslide deformation rate and rainfall and reservoir water level rising.

During stages B1 and D1, the daily average rainfall was 4.11 mm/d and 3.32 mm/d, respectively, with maximum daily rainfall of 42.0 mm and 23.8 mm, and the longest rainfall duration of 3 d and 5 d, respectively (Table 3). There was no significant displacement change observed during this period. During stages A2, C1, C3, and E2, the reservoir water level rising rates were 1.11 m/d, 0.50 m/d, 1.44 m/d, and 0.55 m/d, respectively, with C3 > A2 > E2 > C1. The daily average rainfall was 8.80 mm/d, 12.72 mm/d, 8.69 mm/d, and 9.82 mm/d, respectively, with C1 > E2 > A2 > C3. The maximum daily rainfall was 46.0 mm, 143.4 mm, 74.3 mm, and 102.9 mm, respectively, with C1 > E2 > A2 > C3. The longest rainfall duration was 3 d, 8 d, 5 d, and 5 d, respectively, with C1 > E2 = C3 > A2. The average displacement increment was 4.5 mm, 19.5 mm, 4.0 mm, and 18.5 mm, respectively, with C1 > E2 > A2 > C3. The average displacement rate was 0.50 mm/d, 0.46 mm/d, 0.44 mm/d, and 0.44 mm/d, respectively, with A2 > C1 > E2 = C3 (Table 2). The deformation rate of the landslide was positively correlated with the rainfall intensity, as shown in Fig. 10.

In summary, during stages B1 and D1, the landslide tended to be stable due to the relatively small rainfall intensity. During stages C1 and E2, the rainfall intensity and duration were relatively large, resulting in a significant increase in the average displacement increment. During stage C3, the reservoir water level rising rate was faster than that of A2, but the average displacement rate was smaller (Fig. 10). The analysis indicated that the rising of reservoir water level did not trigger significant deformation of the landslide. During this period, strong rainfall was an important factor in inducing and promoting significant deformation of the landslide. The deformation of the landslide during the reservoir water level rising period was positively correlated with rainfall intensity and duration, with larger deformation occurring with higher rainfall intensity and longer duration.

- The Influence of rainfall on landslide during reservoir water level decline

During stages A3, C2, and C4 (Table 2), the decline rates of reservoir water level were 0.32 m/d, 0.45 m/d, and 0.74 m/d, respectively, with A3 < C2 < C4. The maximum daily rainfall amounts were 48.5 mm, 113.4 mm, and 0.5 mm, with C4 < A3 < C2. The daily average rainfall amounts were 4.28 mm/d, 5.81 mm/d, and 0.03 mm/d, with C4 < A3 < C2. The longest rainfall durations were 3 d, 1 d, and 1 d, with A3 < C2 < C4. The average displacement increments were 10.0 mm, 12.5 mm, and 4.0 mm. The average displacement rates were 0.29 mm/d, 0.57 mm/d, and 0.21 mm/d, with C4 < A3 < C2. During stages B3 and D3 (Table 3), the decline rates of reservoir water level were 0.18 m/d and 0.19 m/d, respectively. The daily average rainfall amounts were 2.47 mm/d and 3.22 mm/d, respectively. The longest rainfall durations were 5 d and 7 d, respectively, with no significant displacement changes observed.

**Figure 11:**
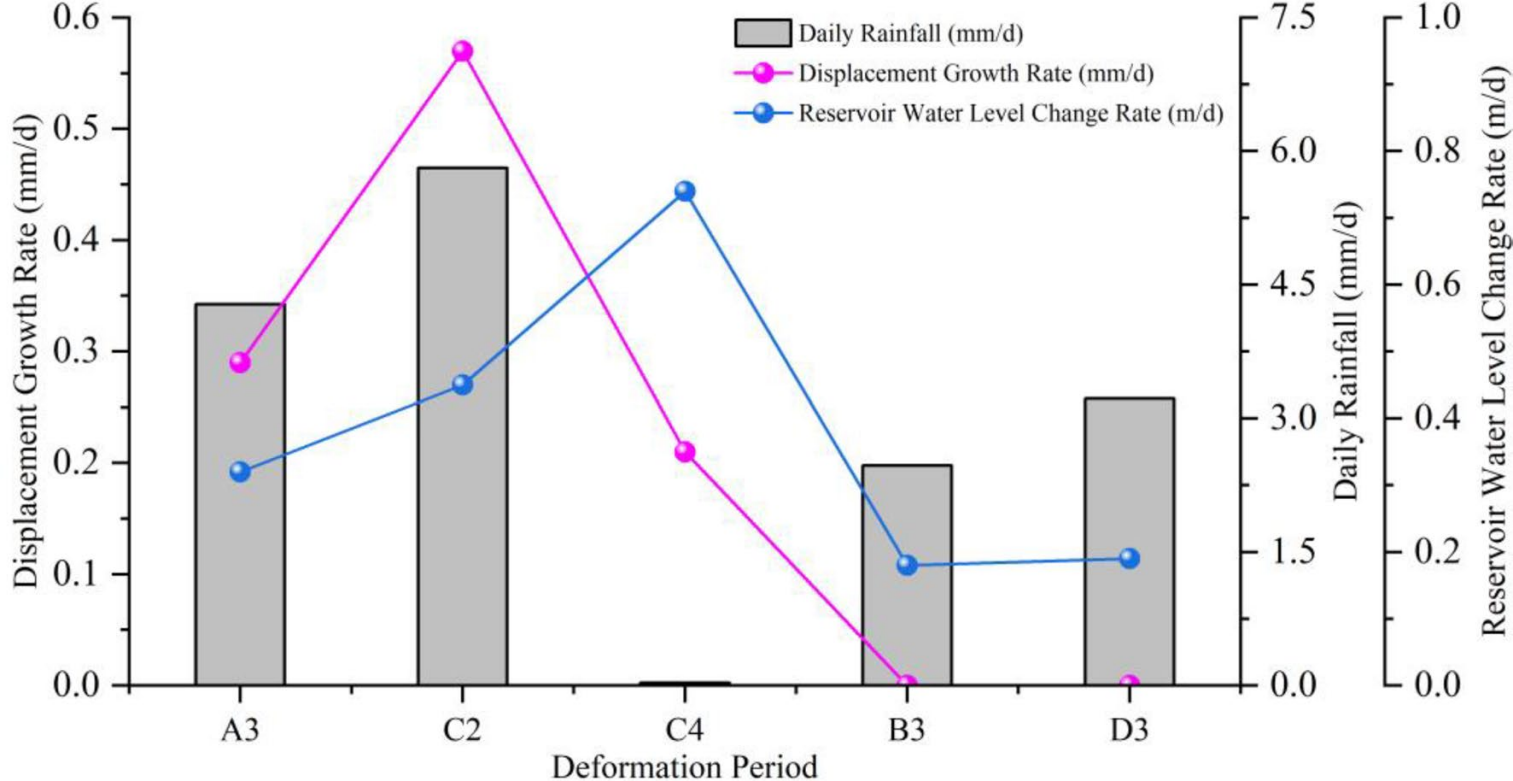
The relationship between landslide deformation rate and rainfall and reservoir water level decline.

In summary, stages B3 and D3 were in a period of slow reservoir water level decline, with low rainfall amounts and no significant displacement changes. Stage C4 had the fastest decline rate of reservoir water level, with little rainfall and small displacement changes. Stage C2 had a cumulative rainfall amount of 127.8 mm, with a maximum daily rainfall amount of 113.4 mm occurring once and large displacement changes. Stage A3 had a cumulative rainfall amount of 145.5 mm, with a maximum daily rainfall amount of 48.5 mm occurring twice and large displacement changes. Therefore, stages C2 and A3 exhibited characteristics of short-term concentrated rainfall, with much larger displacement changes than stage C4. The decline rate of reservoir water level was higher during stage C2 than A3, and the rainfall intensity was also greater during stage C2. However, the average displacement rate was much larger during stage C2 than A3 (Fig. 11).

The analysis indicates that the single factor of slow reservoir water level decline (< 0.3 m/d) will not trigger landslide deformation (Fig. 8). Rapid decline of reservoir water level (> 0.3 m/d) can only induce small displacement changes in landslides, but when combined with intense rainfall, it can trigger larger displacement changes. This suggests that the influence of intense rainfall on landslides is greater than that of rapid decline of reservoir water level. The faster the reservoir water level decline and the greater the rainfall intensity, the more severe the deformation.

- The influence of rainfall on landslide during the low water level operation period

**Figure 12:**
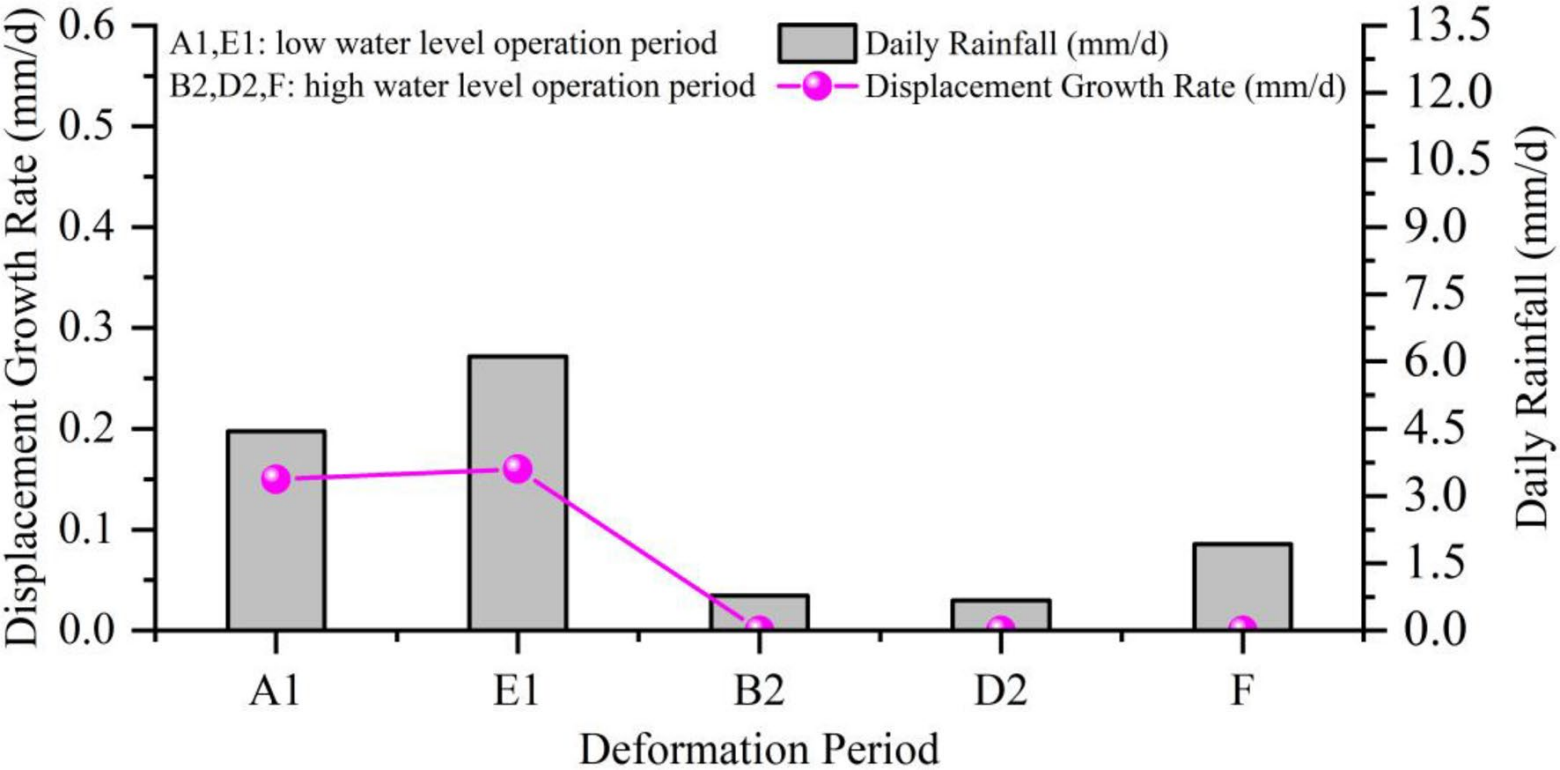
The relationship between landslide deformation rate and rainfall and stable reservoir water level.

Stages A1 and E1 mainly fluctuate in the range of 146–153 m, with average daily rainfall amounts of 4.45 mm/d and 6.11 mm/d, respectively, and E1 > A1; the maximum daily rainfall amounts are 38.5 mm and 64.5 mm, respectively, and E1 > A1; the maximum duration of rainfall are 6 d and 11 d, respectively, and E1 > A1, the average displacement increments are 7.5 mm and 11.5 mm, respectively, the average displacement rates are 0.15 mm/d and 0.16 mm/d, respectively, and E1 > A1 (Table 2, Fig. 12). Therefore, during the low water level operation period, continuous rainfall is an important factor in inducing deformation, and the greater the intensity and duration of the rainfall, the more severe the landslide displacement.

- The influence of rainfall on landslide during the high-water level operation period

During the high-water operation period of the reservoir, the impact of rainfall on landslides in stages B2, D2, and F, where the water level is approximately 175 m, was relatively small. The average daily rainfall during this period was 0.78 mm/d, 0.67 mm/d, and 1.93 mm/d, respectively, resulting in a slow deformation of the landslide (Table 3, Fig. 12).

In summary, the deformation of the Zhangjiacitang landslide is triggered by both heavy rainfall and rapid decline of reservoir water level, but the effect of rainfall on landslide deformation is more pronounced, indicating it as a rainfall-induced landslide. Heavy rainfall can induce and promote landslide deformation at any period, with greater rainfall intensity leading to more severe deformation. Even without heavy rainfall during periods of rapid decline in reservoir water level (> 0.3 m/d), some minor deformation can still be triggered. During periods of slow decline in water level (< 0.3 m/d), as well as during water level rise, low water level operation, and high-water level operation, rainfall is the sole factor causing landslide deformation.

### Mechanism of landslide deformation

The Zhangjiacitang landslide is currently undergoing a continuous development of deformation, but the deformation rate is slow. The main influencing factors of landslide deformation are rainfall and reservoir water level fluctuation. The front edge of the landslide protrudes into the Yangtze River, forming a stepped shape, with some steps being higher, providing favorable and precarious conditions for the formation of the landslide. Changes in reservoir water level cause periodic fluctuations in the internal rock and soil mass, which generate dynamic water pressure. Meanwhile, the river water repeatedly washes and erodes the reservoir bank slope, causing erosion and detachment of the rock and soil mass, loss of lower support in some areas, and reduced resistance to sliding. The material of the landslide body mainly consists of fine-grained sandstone, argillaceous siltstone, and gravel, with poor permeability. Groundwater is not easy to seep out of the landslide body. On the one hand, the rapid decline of reservoir water level causes the groundwater in the slope to lag behind the rate of water level decline, and the water level difference between inside and outside of the slope generates a large dynamic water pressure pointing towards the outside of the slope, triggering landslide deformation. On the other hand, due to the large number of cracks on the surface of the slope and general surface drainage conditions, sustained rainfall causes a large amount of water to be unable to drain in time, leading to saturated soil, decreased shear strength, and a weakened soft layer near the bedrock surface. This results in significant deformation of the landslide.

The fluctuation of reservoir water level and seasonal rainfall have a significant effect on the deformation of the landslide, with the rear part showing more pronounced deformation than the front part, exhibiting the characteristic of a translational landslide along the rock-soil contact surface. Rainfall is the main triggering factor for the landslide, and continuous rainfall results in greater deformation of the middle and rear parts of the landslide, directly pushing the sliding of the front edge of the landslide. The rapid decline of the reservoir water level plays an assisting role, as the deformation of the front edge of the landslide pulls the deformation of the middle and rear parts of the slope.

## Conclusions

1. The Zhangjiacitang landslide exhibits characteristics of translational sliding, with smaller displacement changes observed at the monitoring points near the front of the landslide compared to those near the back. The displacement changes and deformation directions of monitoring points at the front and back of the landslide are generally synchronous, indicating the overall nature of the landslide deformation.
2. The Zhangjiacitang landslide exhibits continuous deformation under the joint action of heavy rainfall and changes in reservoir water level. The deformation of the Zhangjiacitang landslide is more affected by rainfall and is classified as a rainfall-induced landslide. The change of reservoir water level plays a role in promoting landslide deformation, in which the rapid decline of the reservoir water level (> 0.3 m/d) induces minor displacement changes, while slow reservoir water level decline (< 0.3 m/d) and reservoir water level rise do not cause significant deformation. Heavy rainfall can trigger and promote deformation of the landslide at any time. During the reservoir water level rise period, continuous heavy rainfall is the main factor causing deformation of the landslide. During the reservoir water level decline period, short-term heavy rainfall promotes greater deformation of the landslide. The low water level operation period is generally the flood season, with frequent rainfall causing sustained deformation and poor stability of the landslide, while the high-water level operation period is generally the non-flood season, with less rainfall and a more stable landslide. Therefore, according to the actual rainfall and reservoir water level scheduling conditions, the monitoring and inspection efforts in the TGRA can be dynamically adjusted to carry out geological hazard prevention and control more scientifically.

### Supplementary Information

Supplementary Information.

## Data Availability

All data generated or analyzed during this study are included in this published article and its supplementary information files.
